# Supplementary material for: Boosting electrochemical oxygen reduction to hydrogen peroxide coupled with organic oxidation
Source: Nat Commun. 2024 Jul 19;15:6098. doi: 10.1038/s41467-024-50446-2 (PMC11271547; doi:10.1038/s41467-024-50446-2)
Supplement: Supplementary file 1 — Supplementary Information [file 41467_2024_50446_MOESM1_ESM.pdf]

Supplementary Information

Boosting Electrochemical Oxygen Reduction to Hydrogen Peroxide Coupled with Organic Oxidation

Yining Sun[1], Kui Fan[1], Jinze Li[1], Lei Wang[1], Yusen Yang[1,2], Zhenhua Li[1,2], Mingfei Shao[1,2*], Xue Duan[1,2]*

[1]State Key Laboratory of Chemical Resource Engineering, Beijing University of Chemical Technology, Beijing 100029, China
[2]Quzhou Institute for Innovation in Resource Chemical Engineering, Quzhou, 324000, China

*Corresponding author.

E-mail: LZH0307@mail.buct.edu.cn (Z. Li), shaomf@mail.buct.edu.cn (M. Shao)

Supplementary Figures

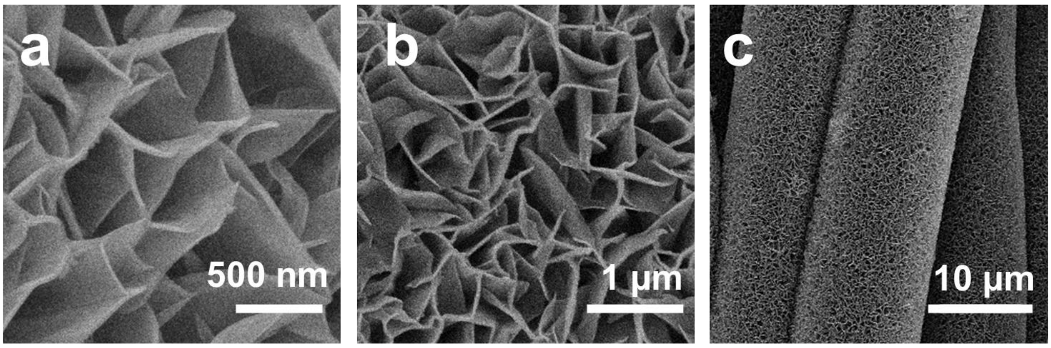

Supplementary Fig. 1 a-c SEM images of NiAl-LDH(MA) at different magnification.

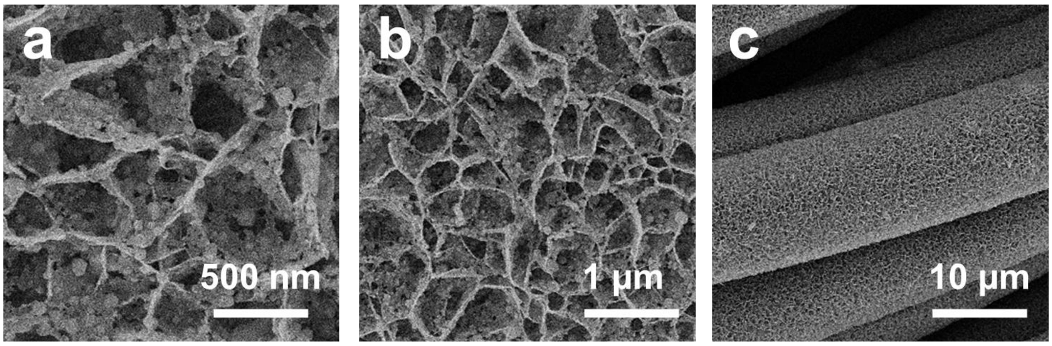

Supplementary Fig. 2 a-c SEM images of Ni-CNS at different magnification.

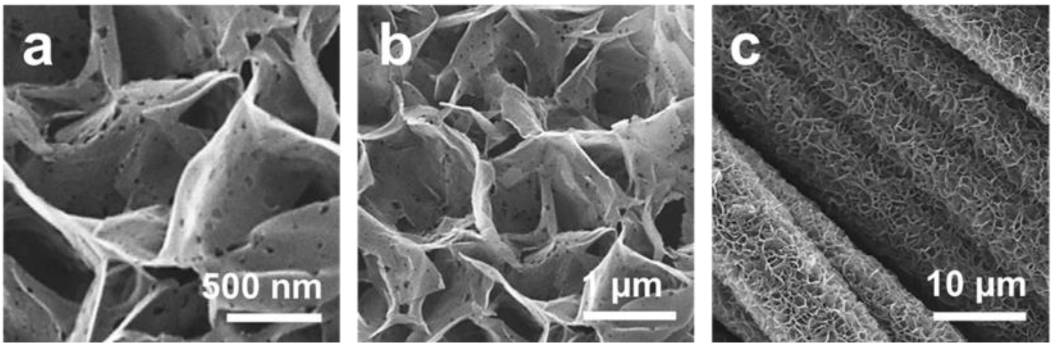

Supplementary Fig. 3 a-c SEM images of CNS at different magnification.

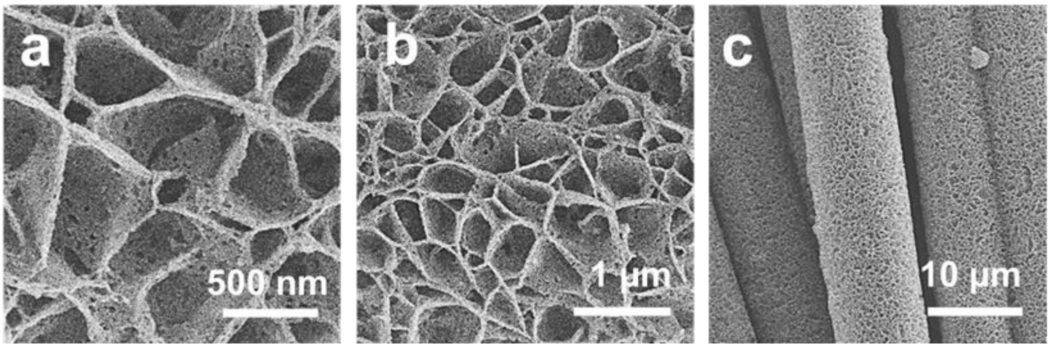

Supplementary Fig. 4 a-c SEM images of Ni-SAC at different magnification.

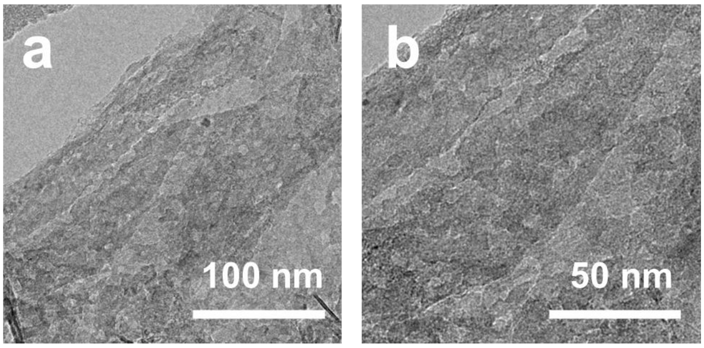

Supplementary Fig. 5 a-b HRTEM images of Ni-SAC at different magnification.

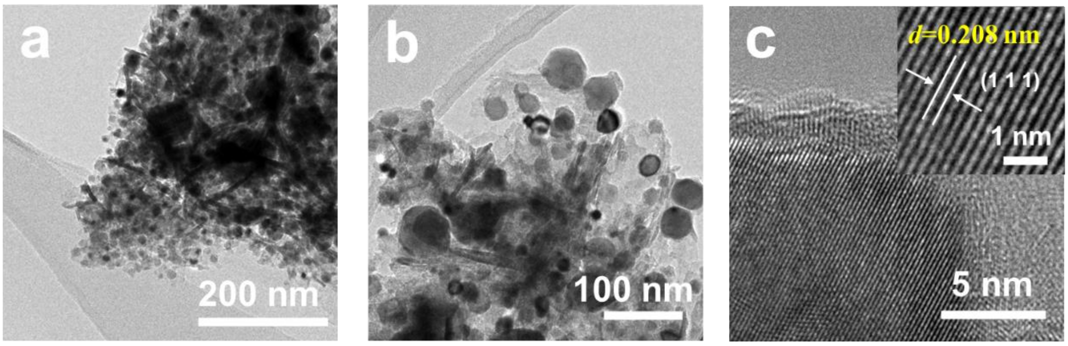

Supplementary Fig. 6 a-c HRTEM images of Ni-CNS at different magnification.

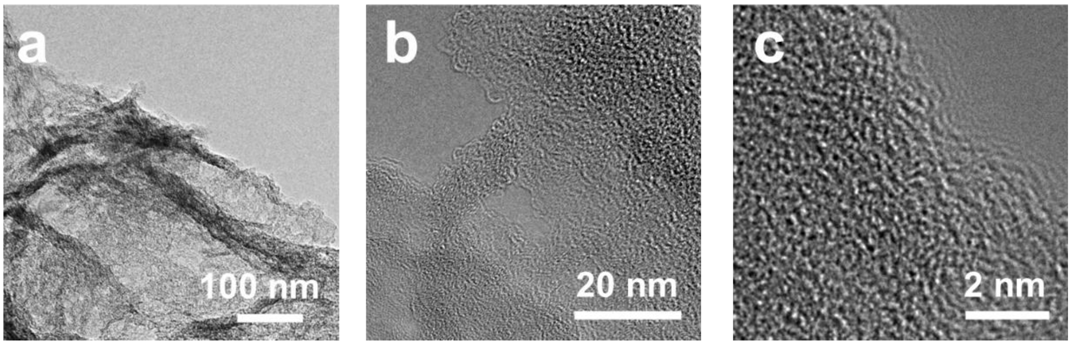

Supplementary Fig. 7 a-c HRTEM images of CNS at different magnification.

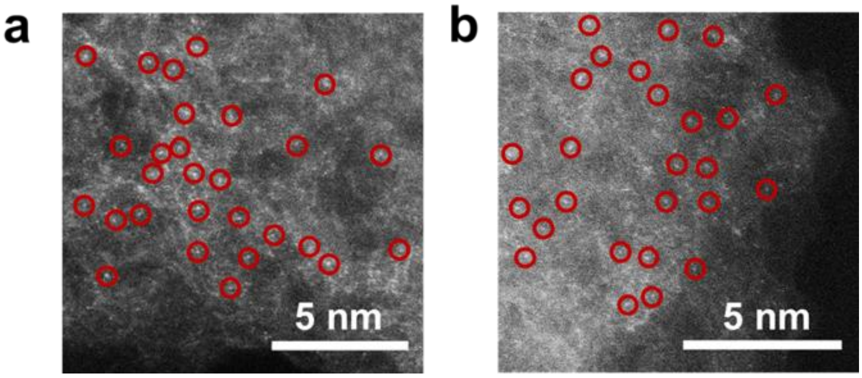

Supplementary Fig. 8 a-b HAADF-STEM image of Ni-SAC at different regions.

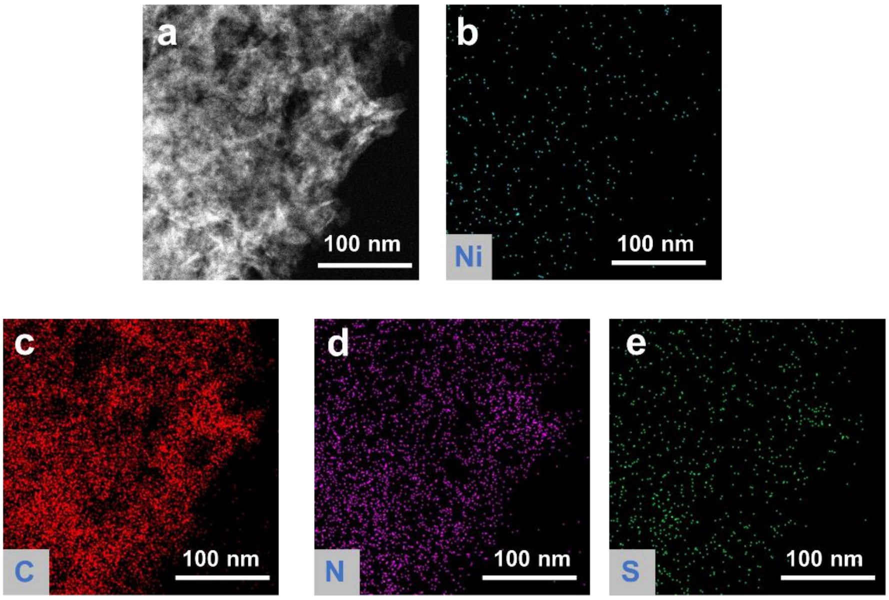

Supplementary Fig. 9 a-e EDX mapping images of Ni-SAC.

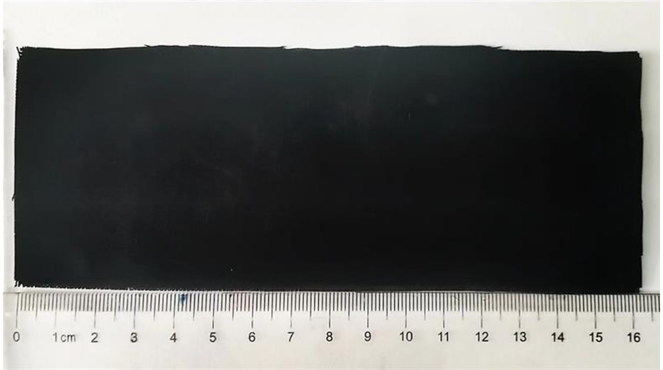

Supplementary Fig. 10 Demonstration of large-area electrocatalyst preparation.

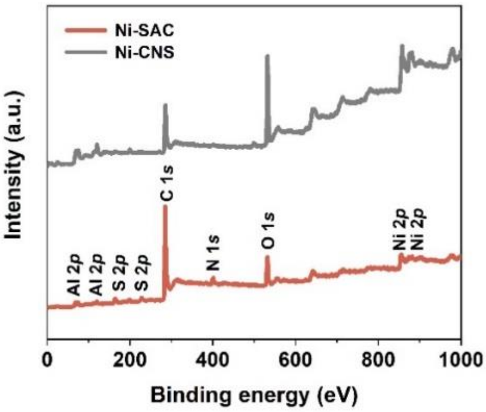

Supplementary Fig. 11 Full XPS spectra of Ni-SAC and Ni-CNS.

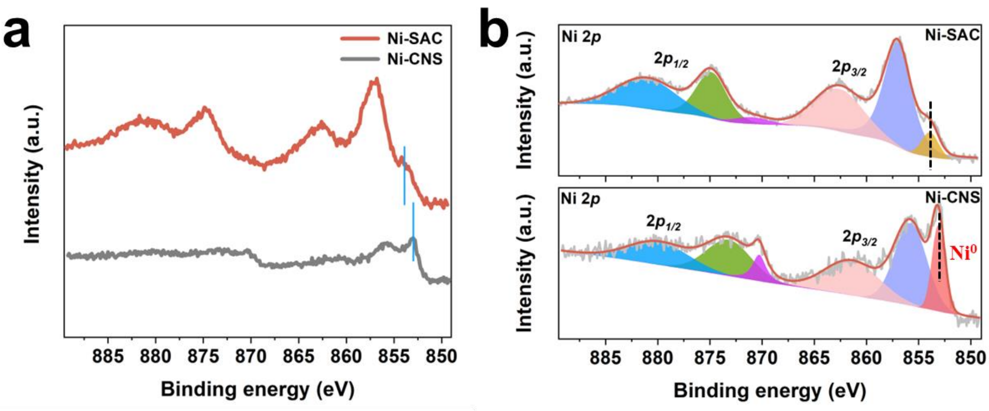

Supplementary Fig. 12 a Ni 2*p* spectra of Ni-CNS and Ni-SAC unfitted after charge correction. **b** Ni 2*p* XPS spectra of Ni-CNS and Ni-SAC.

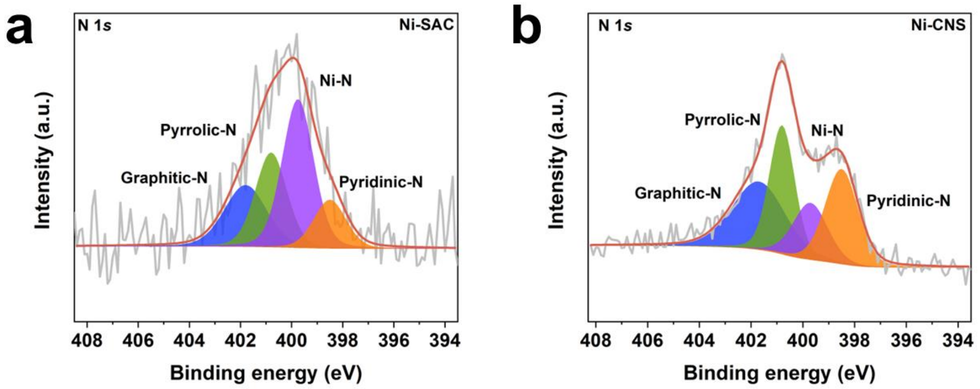

Supplementary Fig. 13 N 1*s* XPS spectra of **a,** Ni-SAC and **b,** Ni-CNS.

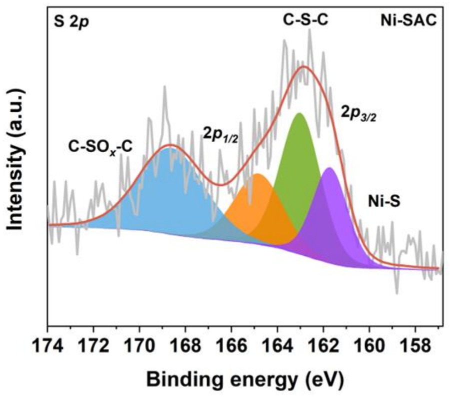

Supplementary Fig. 14 S 2*p* XPS spectra of Ni-SAC.

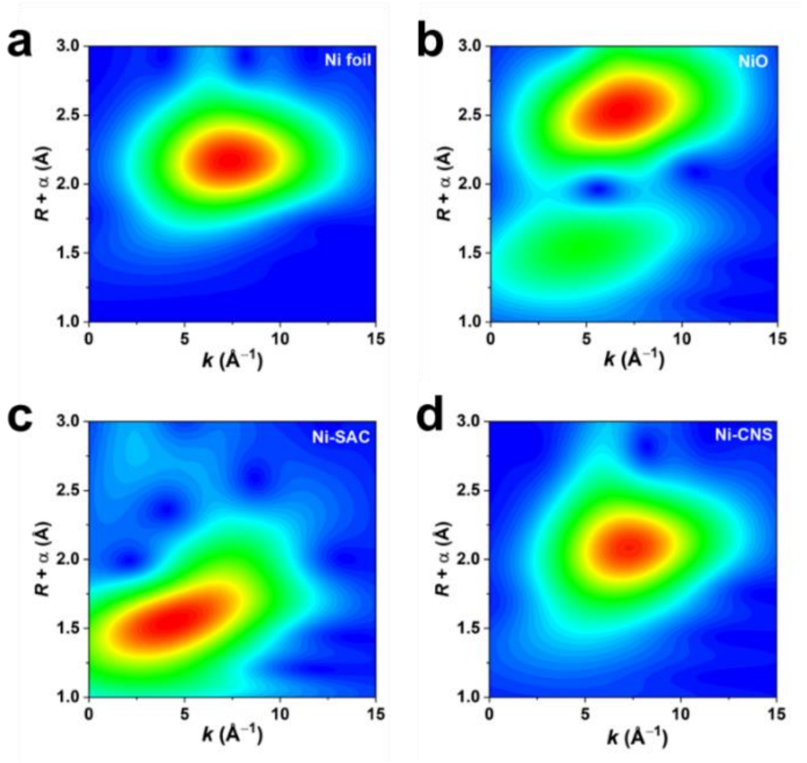

Supplementary Fig. 15 WT-EXAFS spectra of **a,** Ni foil, **b,** NiO, **c,** Ni-SAC and **d,** Ni-CNS.

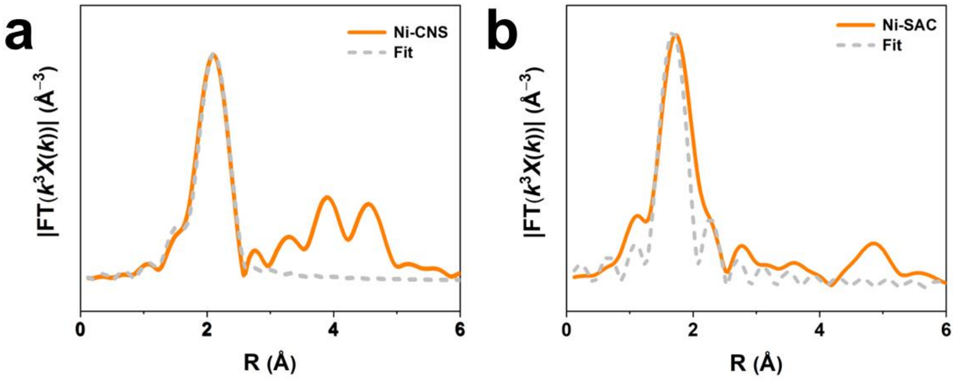

Supplementary Fig. 16 The k^2-weighted Fourier transformations (FT)-EXAFS spectra and FT-EXAFS fitting plots of **a,** Ni-CNS and **b,** Ni-SAC.

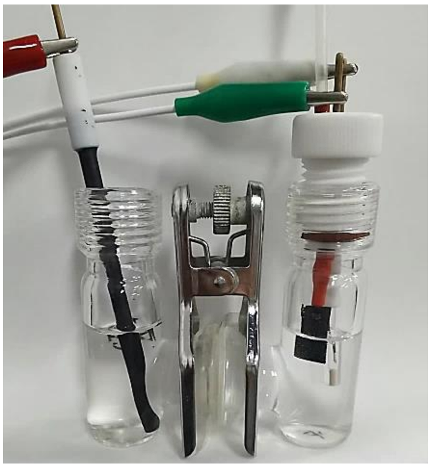

Supplementary Fig. 17 Three-electrode system H-cell device.

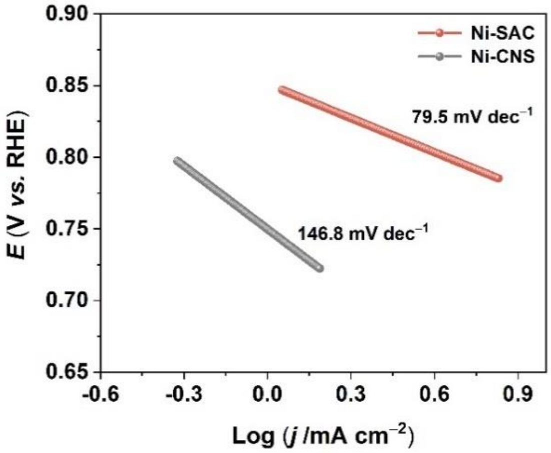

Supplementary Fig. 18 Tafel curves for Ni-SAC and Ni-CNS.

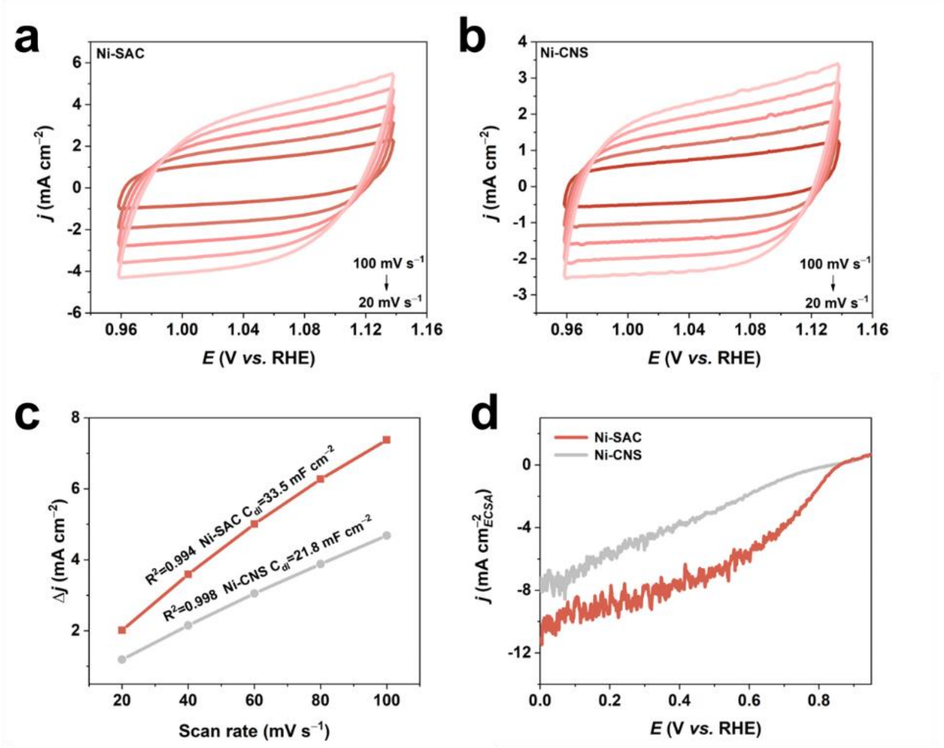

Supplementary Fig. 19 Cyclic voltammetry (CV) of **a,** Ni-SAC and **b,** Ni-CNS in non-Faradaic interval at the scan rates of 20, 40, 60, 80 100mV s^{-1}. **c** Curves of capacitance Δj as a function of different scan rates. **d** LSV curves normalized by ECSA.

$$\mathrm{ECSA} = \frac{C_{dl}}{C_s}$$

C_{dl} is the measured double-layer capacitance, C_s is the specific capacitance of an atomically smooth planar surface, in which the general value of C_s is 0.040 mF cm^{-2}.[1]

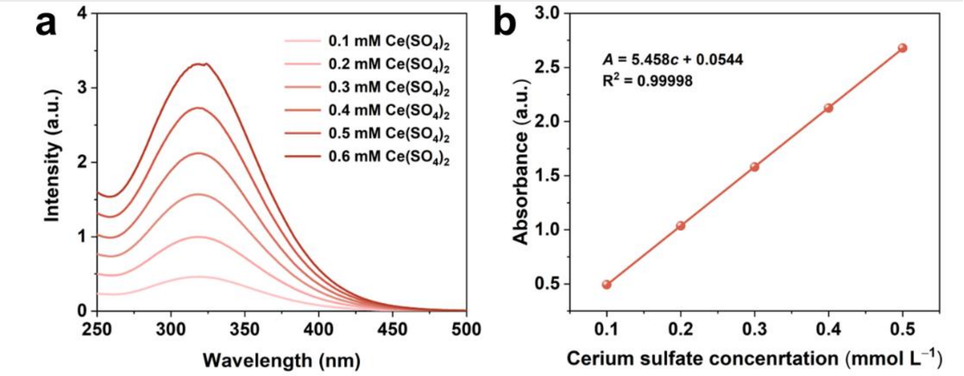

Supplementary Fig. 20 **a** UV-Vis absorption spectra of various $Ce(SO_4)_2$ concentrations. **b** Calibration curve used for calculation the concentration of H_2O_2.

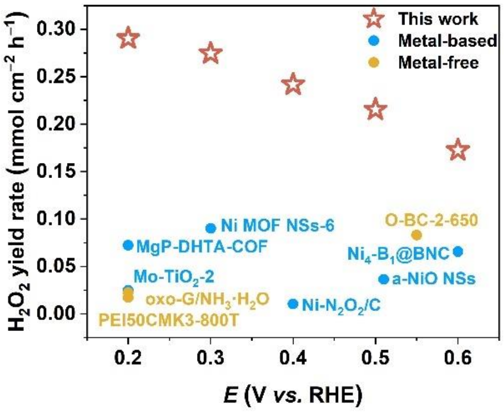

Supplementary Fig. 21 Comparison of the yield rates obtained in H-cell device operated under alkaline conditions for reported work, taking into account different catalyst loadings.

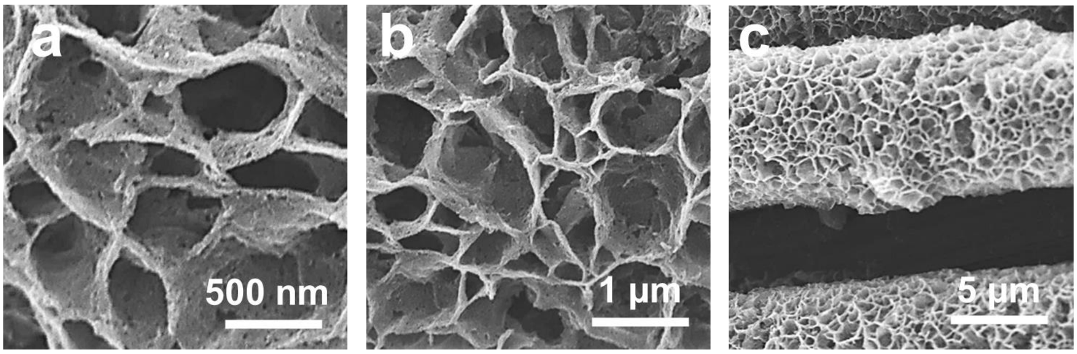

Supplementary Fig. 22 a-c SEM images of Ni-SAC at different magnification after stability test.

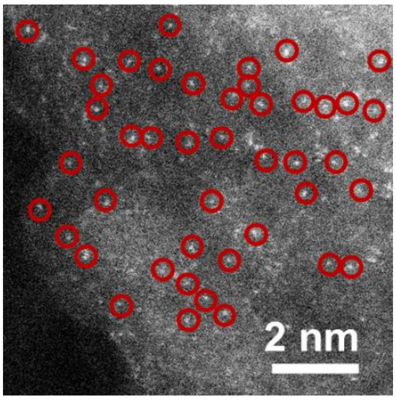

Supplementary Fig. 23 HAADF-STEM images of Ni-SAC after reaction.

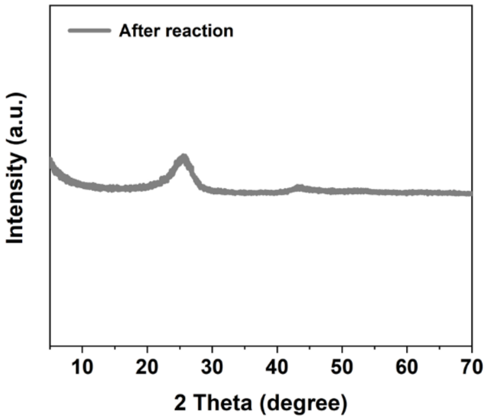

Supplementary Fig. 24 XRD patterns of Ni-SAC after stability test.

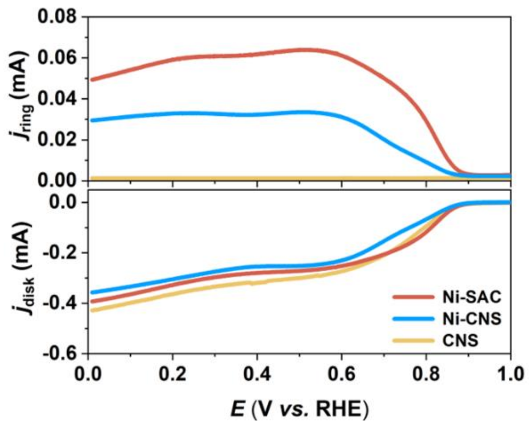

Supplementary Fig. 25 The ORR polarization curves measured by RRDE set-up for Ni-SAC, Ni-CNS, and CNS, respectively.

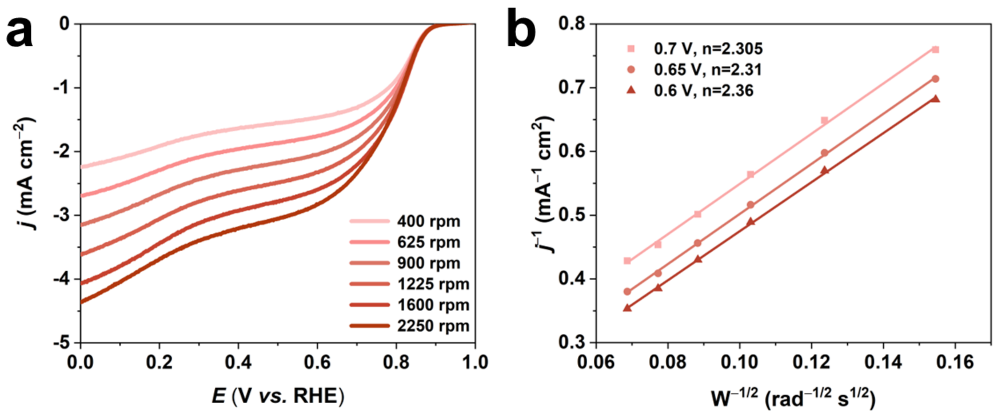

Supplementary Fig. 26 a LSV at different sweep speeds tested on RDE and **b** their corresponding electron transfer numbers calculated by the K-L equation.

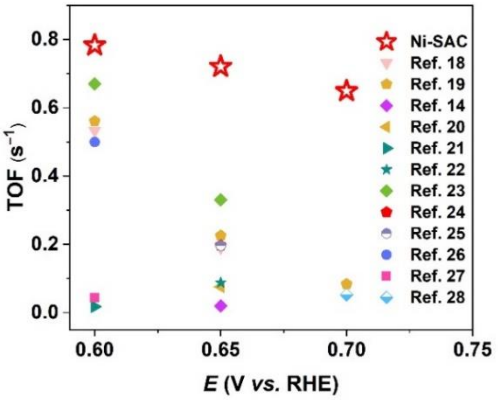

Supplementary Fig. 27 The comparison of TOF values with the currently reported works.

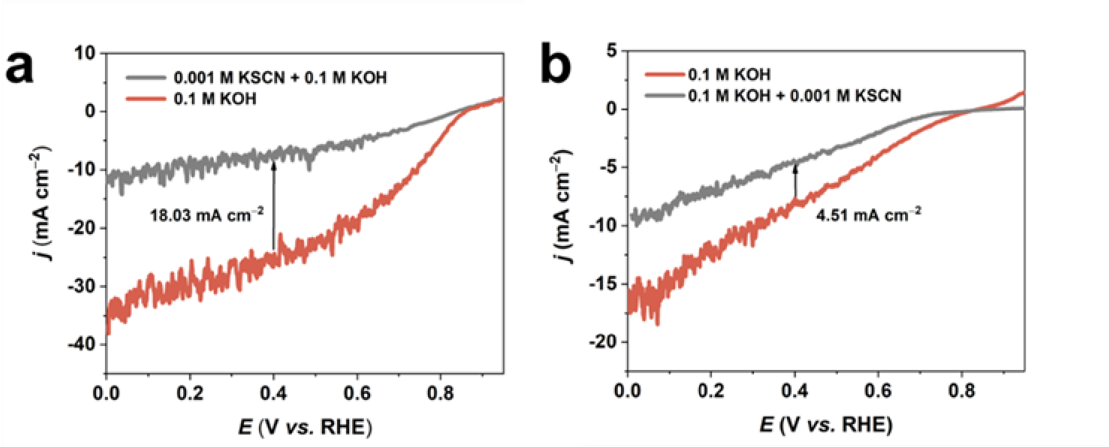

Supplementary Fig. 28 ORR polarization curves before and after 1 mM SCN^- poisoning in 0.1 M O_2-saturated KOH for **a,** Ni-SAC and **b,** Ni-CNS.

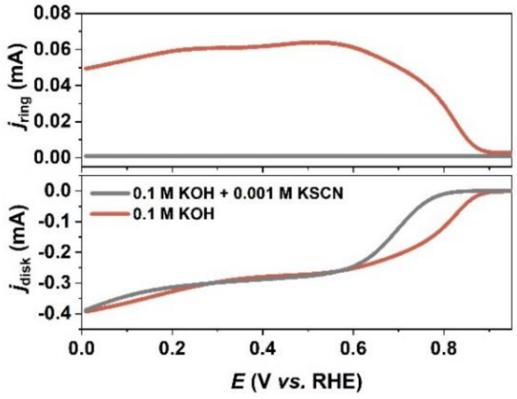

Supplementary Fig. 29 The LSV curves measured on RRDE after poisoning with 0.001 M KSCN.

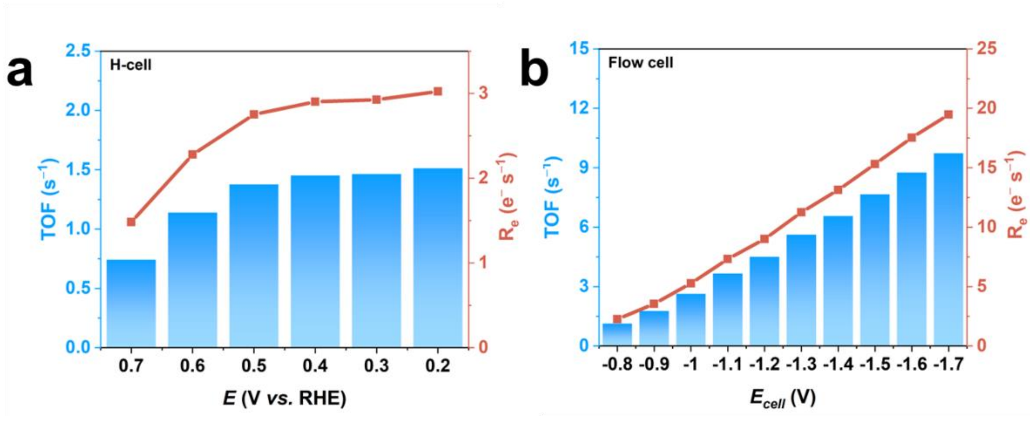

Supplementary Fig. 30 TOF values and R_e of Ni-SAC at different voltages in **a,** H-cell and **b,** flow cell.

R_e is defined as the total number of electrons consumed per second per the number active sites, which for the $2e^-$ ORR reaction is defined as:

$$R_e = 2 \times \text{TOF}$$

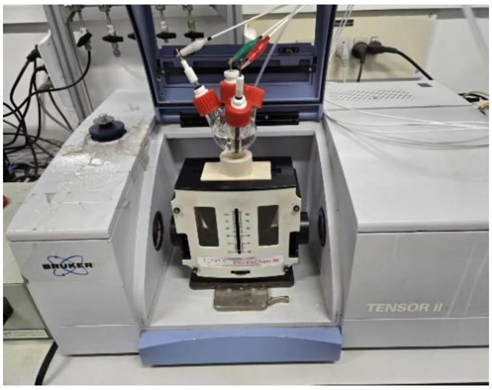

Supplementary Fig. 31 *In-situ* FTIR device diagram.

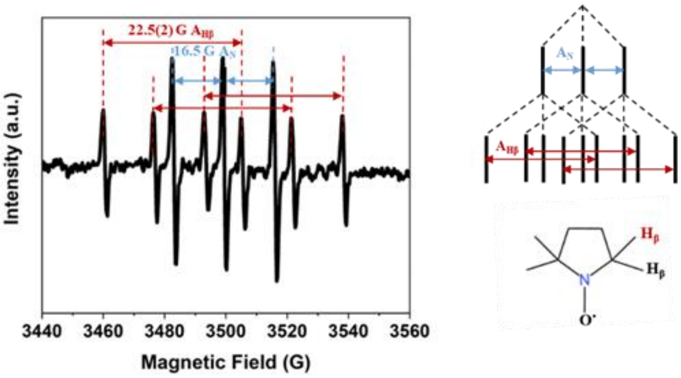

Supplementary Fig. 32 HFS analysis of hydrogen radicals and corresponding nonuple peaks.

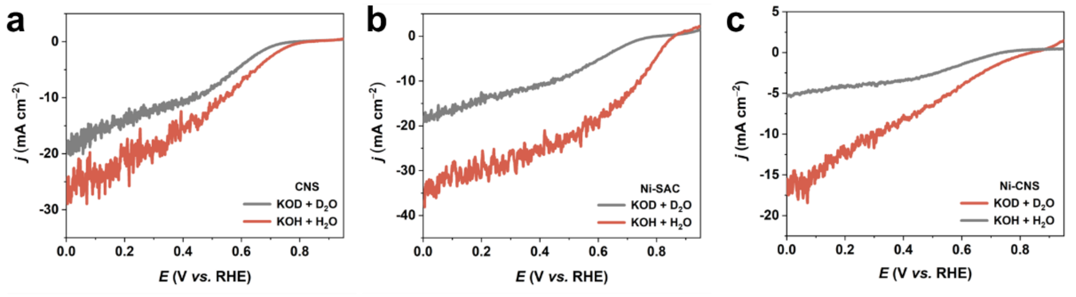

Supplementary Fig. 33 The LSV curves of **a,** CNS, **b,** Ni-SAC and **c,** Ni-CNS in O_2-saturated 0.1 M KOH/0.1 M KOD solutions.

The KIE value is calculated by comparing the ratio of j_{H2O} to j_{D2O} at 0 V.

$$\mathrm{KIE}_{H/D} = \left[\frac{K_{H_2O}}{K_{D_2O}}\right]\mathrm{E} = \left[\frac{j_{H_2O}}{j_{D_2O}}\right]\mathrm{E}$$

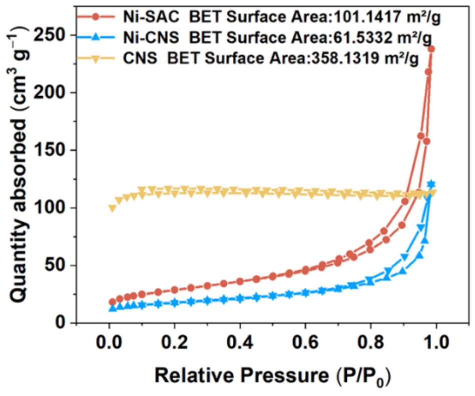

Supplementary Fig. 34 N_2 sorption isotherms of Ni-SAC, Ni-CNS, CNS.

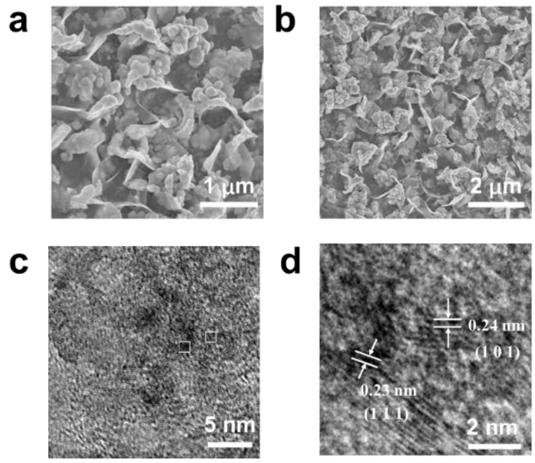

Supplementary Fig. 35 a-b SEM images of Au/Ni$(OH)_2$ at different magnification. **c-d** HRTEM images of Au/Ni$(OH)_2$ at different magnification.

The SEM images show the Au nanoparticles were uniformly loaded on the Ni$(OH)_2$ nanosheets. The HRTEM results further displays that the lattice fringes that can be assigned to the (101) plane of *β*-Ni$(OH)_2$ and the (111) plane of Au, respectively, affirming the successful synthesis of Au/Ni$(OH)_2$.

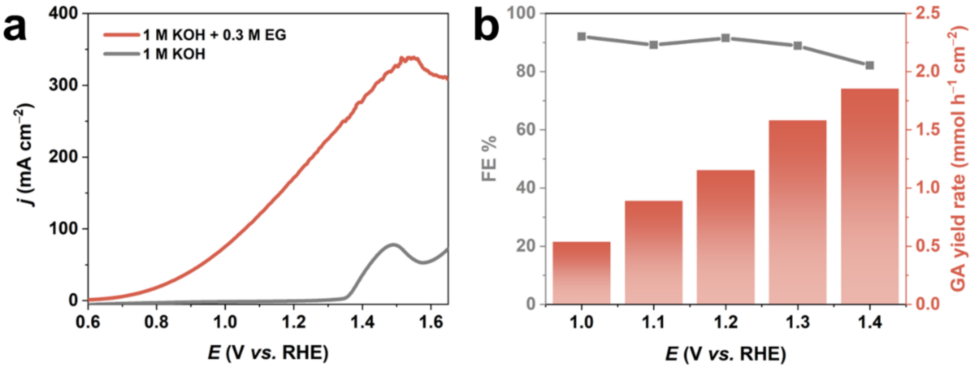

Supplementary Fig. 36 a LSV curves of EG oxidation in 1 M KOH and 1 M KOH+ 0.3 EG of three-electrode system. **b** FE and yield rate of GA in the 1.0 V−1.4 V potential range.

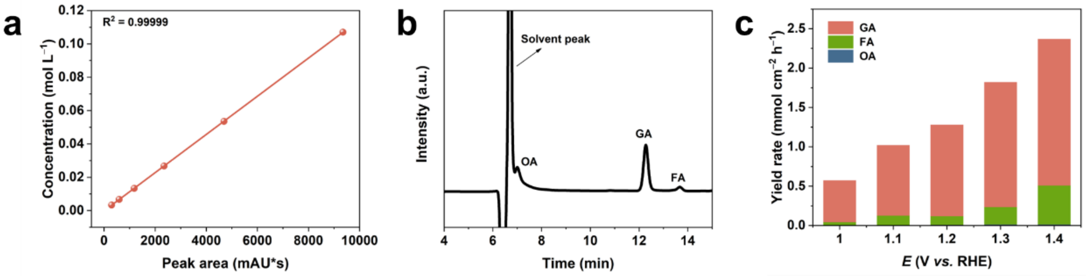

Supplementary Fig. 37 a Standard curve for glycolic acid. **b** HPLC chromatogram of EG electrooxidation products over Au/Ni(OH)$_2$ in 1 M KOH + 0.3 M EG at 1.0 V for 1 h (OA and FA represent for oxalic acid and formic acid, respectively). **c** Product distribution of EG electrooxidation in the voltage range 1.0-1.4 V.

We present the liquid phase data related to the EG electrooxidation reaction, thus further illustrating the production capacity of Au/Ni(OH)$_2$ for GA. Specifically, we provide the standard curve used to calculate the concentration of GA and identify the peaks attributed to the EG electrooxidation products in High Performance Liquid Chromatography (HPLC) chromatogram **(Supplementary Figs. 37a-b)**. Subsequently, we further give the product distribution of EG electrooxidation in the potential range of 1.0-1.4 V, where GA is the main product while OA and FA are the by-products **(Supplementary Fig. 37c)**.

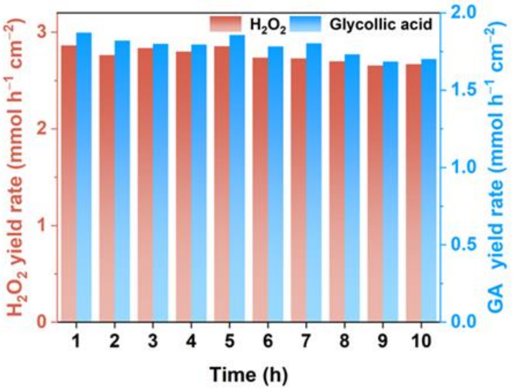

Supplementary Fig. 38 Stability test at −1.7 V for 10 h.

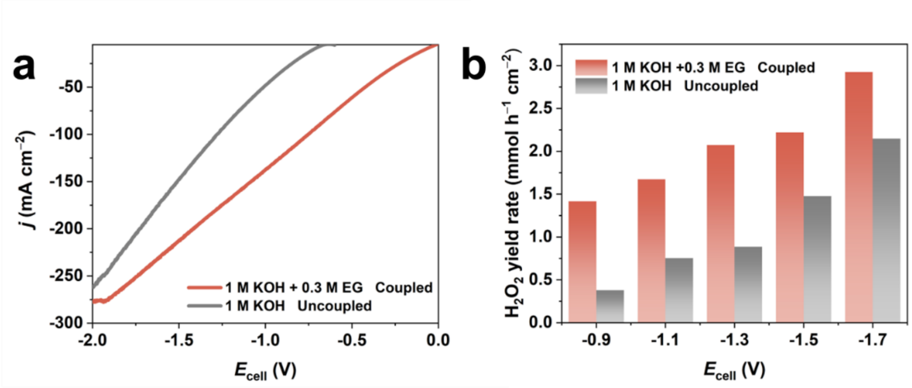

Supplementary Fig. 39 **a** Comparison of LSV curves between uncoupled systems (NiFe-LDH as anode) and coupled systems (Au/Ni(OH)$_2$ as anode). **b** Comparison of H_2O_2 yield rate between the above two systems.

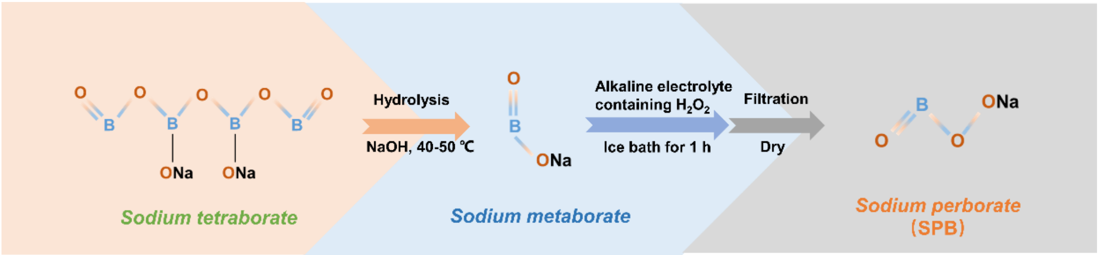

Supplementary Fig. 40 SPB synthesis process.

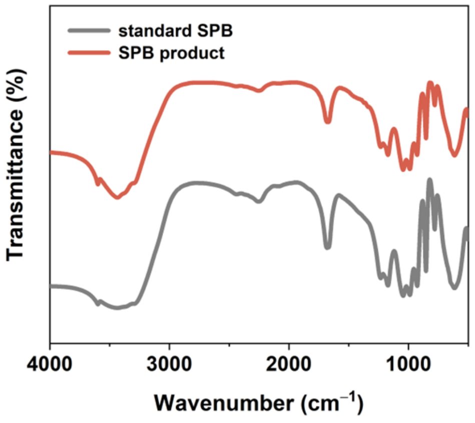

Supplementary Fig. 41 FTIR spectra of standard SPB and SPB product.

Supplementary Note 1

In terms of turnover frequency (TOF) value, which evaluates the difference in intrinsic activity by taking into account the difference in active site density of the catalyst[2, 3]. For the $2e^-$ORR reaction, the TOF value is defined as

$$\text{TOF}\ \left(s^{-1}\right) = \frac{\text{number of } O_2 \text{ turnover}}{\text{number of active sites}} = \frac{j}{2nF}$$

j represents the current density of H_2O_2 production, *F* is the Faraday constant (96485 C mol^{-1}), *n* is the number of active sites, *j*/2*F* represents for the number of O_2 turnover.

$$O_2 \text{ turnover} = j \left[\frac{\text{mA}}{\text{cm}^2}\right] * \frac{1\left[\frac{\text{C}}{\text{s}}\right]}{1000[\text{mA}]} * \frac{1\left[\text{mol e}^-\right]}{96485\ [\text{C}]} * \frac{1[\text{mol } O_2]}{2\left[\text{mol e}^-\right]} *6.02*10^{23} \left[\frac{\text{atom } O_2}{\text{mol } O_2}\right]$$

$$\text{number of active sites} = L \left[\frac{\text{mg}}{\text{cm}^2}\right] * R[\text{wt }\%]* \frac{1[\text{mmol}]}{\text{W [mg]}} *6.02*10^{20} \left[\frac{\text{atom}O_2}{\text{mmol}}\right]$$

L is the amount of catalyst loaded on the electrode, *R* is the weight fraction, and W is the atomic weight of the corresponding element of active sites.

Supplementary Note 2

ESR serves as a technique for the identification of radical species by detecting relatively long-lived spin adducts (Typically nitrogen oxides, characterized by resonance stabilization attributable to their unpaired electrons) obtained by reacting radicals with diamagnetic compounds (spin traps, in this case, DMPO)[4]. The collected spectra were subsequently analyzed for hyperfine splitting (HFS) parameters as well as the magnitude of the splitting to identify the species of the captured radicals. We can identify the nonuple peaks with an intensity ratio of 1:1:2:1:2:1:2:1:1, which hyperfine coupling constants are A_N = 16.5 G and $A_{H\beta}$ = 22.5 G (Supplementary Fig. 38)[5, 6]. The

above results all correspond to DMPO-H adducts. Therefore, we refer to the active species captured by DMPO as active hydrogen.

Supplementary Note 3

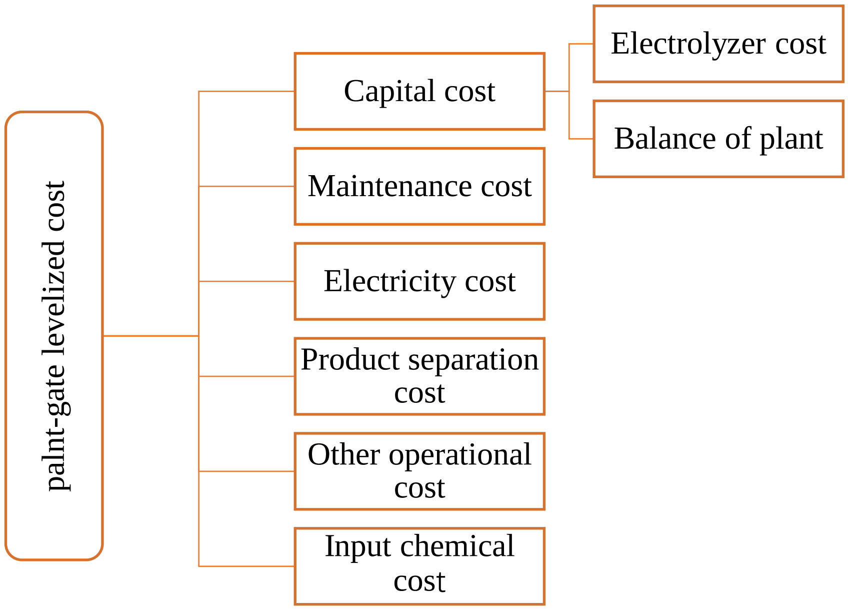

The cost/revenue of coupled and uncoupled systems is assessed in techno-economic evaluation with the cell voltage of −1.7 V as an example.

Some assumptions of Techno-economic evaluation (TEA) are as follows:

1. Electrolysis area is 500 m^2 according to industrial demand.
2. The membrane cost is 180 \$ m^{-2}.
3. The installation factor is 1.2 and the stack cost is 460 \$ kW^{-1}.
4. The power conversion efficiency is 90% and the price of renewable electricity is 5 ¢ kWh^{-1}.
5. 350 days/year of operation time
6. Maintenance cost is 2.5% of the capital cost.
7. Water and electrolyte costs are 30% of raw materials.

8. Balance of cost is 35% of the cost of electrolyzer system.
9. Other operational costs are 20% of the raw materials.
10. Product separation cost is 60% of the raw materials.

Uncoupled system:

The current density and *FE* are 194.048 mA cm^{-2} and 79% at −1.7 V cell voltage, respectively.

The total current is calculated as:

$$\text{Current(A)} = \text{Electrolyzer area}(\text{m}^2) \times \text{Current density}\,(\frac{\text{A}}{\text{m}^2})$$

$$= 0.194048\ \text{mA cm}^{-2} \times 500\ \text{m}^2 \times \frac{10000\text{cm}^2}{\text{m}^2} = 970240\ \text{A}$$

H_2O_2 yield rate (kg/hr):

$$\frac{\text{I} \times \text{M} \times \text{t} \times \text{FE}}{\text{n} \times \text{F} \times 1\ \text{hr}} = \frac{970240\ \text{A} \times 34\frac{\text{g}}{\text{mol}} \times 3600\ \text{s}}{2 \times 96485\frac{\text{C}}{\text{mol}} \times 1\ \text{hr}} = 615.42\ \text{kg/hr}$$

O_2 consumption rate:

$$\frac{\text{m}_{\text{H2O2}} \times \text{M}_{\text{O2}}}{\text{M}_{\text{H2O2}}} = \frac{615.42\frac{\text{kg}}{\text{hr}} \times \frac{32\text{g}}{\text{mol}}}{34\frac{\text{g}}{\text{mol}}} \times \frac{1}{0.79} = 733.19\ \text{kg/hr}$$

Anode (OER) production O_2 rate:

$$\frac{\text{I} \times \text{M} \times \text{t} \times \text{FE}}{\text{n} \times \text{F} \times 1\ \text{hr}} = \frac{970240\ \text{A} \times 32\frac{\text{g}}{\text{mol}} \times 3600\ \text{s}}{4 \times 96485\frac{\text{C}}{\text{mol}} \times 1\ \text{hr}} = 289.61\text{kg/hr}$$

Input chemicals:

$$\text{O}_2\ \text{cost} = 733.19\frac{\text{kg}}{\text{hr}} \times 24\ \text{hr} \times 350\ \text{days} \times 0.092\frac{\$}{\text{kg}} = 566609.23\ \$$$

Product revenue:

$$H_2O_2 \text{ revenue} = 615.42\frac{\text{kg}}{\text{hr}} \times 24 \text{ hr} \times 350 \text{ days} \times 1.087\frac{\$}{\text{kg}} = 5619276.94 \ \$$$

$$O_2 \text{ revenue (anode)} = 289.61\frac{\text{kg}}{\text{hr}} \times 24 \text{ hr} \times 350 \text{ days} \times 0.092\frac{\$}{\text{kg}} = 223810.61 \ \$$$

Electrolyzer cost:

$$\frac{460\$}{\text{kW}} \times 0.194048\frac{\text{A}}{\text{cm}^{-2}} \times 1.7 \text{ V} \times \frac{10^4\text{cm}^2}{\text{m}^2} \times \frac{\text{kW}}{1000\text{W}} \times 500 \text{ m}^2 \times 1.2 = 910473.22 \ \$$$

Balance of plant cost:

$$910473.22 \ \$ \times \frac{0.35}{0.65} = 490254.81 \ \$$$

Electricity cost:

$$\text{Power} = \text{U} \times \text{I} = 1.7 \text{ V} \times 970240 \text{ A} = 1649.408 \text{ kW}$$

$$1649.408 \text{ kW} \times 0.05\frac{\$}{\text{kWh}} \times 350 \times 24 \text{ hr} \times \frac{1}{0.9} = 769723.73 \ \$$$

Maintenance cost:

$$1400728.0246 \times 2.5\% = 35018.2 \ \$$$

Membrane cost:

$$180\frac{\$}{\text{m}^2} \times 500 \text{ m}^2 = 90000 \ \$$$

Other cost:

$$566609.23 \ \$ \times 20\% = 113321.85 \ \$$$

Water and electrolyte cost:

$$566609.23 \times 30\% = 169982.77 \ \$$$

Coupled system:

The optimal *FE* at −1.7 V cell voltage is 90%, and the current density can reach

243.684 mA cm^{-2}.

Total current:

$$\text{Current(A)} = \text{Electrolyzer area}(\text{m}^2) \times \text{Current density}\ (\frac{\text{A}}{\text{m}^2})$$

$$= 0.243684\ \text{mA cm}^{-2} \times 500\ \text{m}^2 \times \frac{10000\text{cm}^2}{\text{m}^2} = 1218421\ \text{A}$$

H_2O_2 yield rate (kg/hr):

$$\frac{\text{I} \times \text{M} \times \text{t} \times \text{FE}}{\text{n} \times \text{F} \times 1\ \text{hr}} = \frac{1218421\ \text{A} \times 34\frac{\text{g}}{\text{mol}} \times 3600\ \text{s}}{2 \times 96485\frac{\text{C}}{\text{mol}} \times 1\ \text{hr}} = 772.84\ \text{kg/hr}$$

O_2 consumption rate:

$$\frac{m_{H2O2} \times M_{O2}}{M_{H2O2}} = \frac{772.84\frac{\text{kg}}{\text{hr}} \times \frac{32\text{g}}{\text{mol}}}{34\frac{\text{g}}{\text{mol}}} \times \frac{1}{0.9} = 808.20\ \text{kg/hr}$$

GA yield rate (kg/hr):

$$\frac{\text{I} \times \text{M} \times \text{t} \times \text{FE}}{\text{n} \times \text{F} \times 1\ \text{hr}} = \frac{1218421\text{A} \times 76.05\frac{\text{g}}{\text{mol}} \times 3600\ \text{s}}{4 \times 96485\frac{\text{C}}{\text{mol}} \times 1\ \text{hr}} = 864.33\ \text{kg/hr}$$

EG consumption rate:

$$\frac{m_{GA} \times M_{EG}}{M_{GA}} = \frac{\frac{864.33\text{kg}}{\text{hr}} \times 62.07\frac{\text{g}}{\text{mol}}}{\frac{76.05\text{g}}{\text{mol}}} \times \frac{1}{0.9} = 783.83\ \text{kg/hr}$$

Input chemicals:

$$O_2\ \text{cost} = 808.20\frac{\text{kg}}{\text{hr}} \times 24\ \text{hr} \times 350\ \text{days} \times 0.092\frac{\$}{\text{kg}} = 624576.96\ \$$$

$$\text{EG cost} = 783.83\frac{\text{kg}}{\text{hr}} \times 24\ \text{hr} \times 350\ \text{days} \times 0.59\frac{\$}{\text{kg}} = 3884661.48\ \$$$

Product revenue:

$$H_2O_2\ \text{revenue} = 772.84\frac{\text{kg}}{\text{hr}} \times 24\ \text{hr} \times 350\ \text{days} \times 1.087\frac{\$}{\text{kg}} = 7056647.47\ \$$$

$$\text{GA revenue (anode)} = 864.33\frac{\text{kg}}{\text{hr}} \times 24\ \text{hr} \times 350\ \text{days} \times 2.83\frac{\$}{\text{kg}} = 20546852.76\ \$$$

Electrolyzer cost:

$$\frac{460\$}{\text{kW}} \times 0.243684\frac{\text{A}}{\text{cm}^{-2}} \times 1.7\ \text{V} \times \frac{10^4\text{cm}^2}{\text{m}^2} \times \frac{\text{kW}}{1000\text{W}} \times 500\text{m}^2 \times 1.2 = 1143366.27\ \$$$

Balance of plant cost:

$$1143366.27\ \$ \times \frac{0.35}{0.65} = 615658.77\ \$$$

Electricity cost:

$$\text{Power} = \text{U} \times \text{I} = 1.7\ \text{V} \times 1218421\ \text{A} = 2071.316\ \text{kW}$$

$$2071.316\ \text{kW} \times 0.05\frac{\$}{\text{kWh}} \times 350 \times 24\ \text{hr} \times \frac{1}{0.9} = 966614.13\ \$$$

Maintenance cost:

$$1759025.04 \times 2.5\% = 43975.63\ \$$$

Membrane cost:

$$180\frac{\$}{\text{m}^2} \times 500\ \text{m}^2 = 90000\ \$$$

Other cost:

$$4509238.44\ \$ \times 20\% = 901847.69\ \$$$

Water and electrolyte cost:

$$4509238.44\ \$ \times 30\% = 1352771.53\ \$$$

Separation cost (anode):

$$3884661.48\ \$ \times 60\% = 2330796.89\ \$$$

Supplementary Tables

Supplementary Table 1. Quantification of Ni species in the as prepared electrocatalysts by ICP-OES.

**Samples**	**Ni-SAC**	**Ni-CNS**	**CNS**
Content of Ni (wt. %)	1.16%	8.66%	0.0026%

Supplementary Table 2. Ni species content detect by XPS.

**Samples**	**Ni-SAC**	**Ni-CNS**
Content of Ni (wt. %)	1.59%	9.32%

Supplementary Table 3. Specific information on XPS data fitting.

Sample	XPS Peak		Position (eV)	Area	FWHM (eV)
Ni-SAC	Ni 2*p*	Ni $2p_{3/2}$	853.9	2026.386	1.937
			857.0	16666.620	3.124
		Ni $2p_{1/2}$	871.0	1142.627	3.969
			874.9	7564.777	3.479
	N 1*s*	pyridinic-N	398.5	137.783	1.489
		Ni-N	399.8	408.814	1.451
		pyrrolic-N	400.8	261.671	1.459
		graphitic-N	401.8	228.294	1.951
	S 2*p*	Ni-S	161.7	240.370	1.817
		C-S-C $2p_{3/2}$	163.0	407.669	2.091
		C-S-C $2p_{1/2}$	164.8	245.341	2.508
		C-SO_x-C	168.6	405.683	3.398
Ni-CNS	Ni 2*p*	Ni $2p_{3/2}$	853.1	1825.358	1.434
			855.8	4413.683	3.608
		Ni $2p_{1/2}$	870.3	834.994	1.542
			873.2	2814.490	5.075
	N 1*s*	pyridinic-N	398.5	652.840	1.508
		Ni-N	399.7	410.720	1.584
		pyrrolic-N	400.8	683.506	1.164
		graphitic-N	401.7	750.613	2.417

Supplementary Table 4. Curve-fitting results of Ni K-edge EXAFS spectra.

Sample	Shell	*R* (Å)	*CN*	σ^2(10^{-3}Å^2)	ΔE^0(eV)	*R* factor (%)
Ni-SAC	Ni–N/S	2.06	3.9	3.5	8.5	0.9
Ni-CNS	Ni–N/S	2.01	4.9	1.6	8.3	1.1
	Ni–Ni	2.48	5.6	6.4		
Ni-foil	Ni–Ni	2.48	11.5	6.0	6.1	0.1
NiO	Ni–O	2.07	5.4	7.5	3.1	0.6
	Ni–Ni	2.95	11.0	7.5		

CN is the coordination number; *R* is the distance between absorber and backscatter atoms; σ^2 is Debye-Waller factor value; ΔE_0 is inner potential correction accounts for the difference in the inner potential between the sample and the reference compound; R-factor indicates the goodness of the fit. Fitting range: $2.5 \leq k$ (/Å) ≤ 11.0 and $1.0 \leq R$ (Å) ≤ 3.

Supplementary Table 5. Comparison of 2e^{-} ORR performance in H-cell under alkaline conditions (0.1 M KOH) with currently reported work.

**No.**	**Catalyst**	**Yield rate (mmol cm^{-2} h^{-1})**	**Yield rate (mmol $g_{cat}$$^{-1}$h^{-1})**	**Ref.**
1	Co-SAs/NC	/	38.1 (@0.5 V)	7
2	Mo-TiO_2-2	~0.025 (@0.2 V)	~250 (@0.2 V)	8
3	Oxo-G/NH_3·H_2O	0.02248 (@0.2 V)	224.8 (@0.2 V)	9
4	Ni_4-B_1@BNC	0.0655 (@0.6 V)	128.5 (@0.6 V)	10
5	PEI50CMK3_800T	0.0173 (@0.2 V)	345.5 (@0.2 V)	11
6	Ni MOF NSs-6	0.09 (@0.3 V)	90 (@0.3 V)	12
7	O-BC-2-650	~ 0.083 (@0.55 V)	412.8 (@0.55 V)	13
8	Ni-N_2O_2/C	~ 0.0104 (@0.4 V)	45.1 (@0.4 V)	14
9	a-NiO NSs	0.03625 (@0.51 V)	145 (@0.51 V)	15
10	N-DCDs	/	613.58 (@0.3 V)	16
11	MgP-DHTA-COF	0.0724 (@0.2 V)	362 (@0.2 V)	17
**12**	**Ni-SAC**	**0.29±0.02 (@0.2 V)**	**726.1 (@0.2 V)**	**This work**

Supplementary Table 6. Comparison of TOF values of Ni-SAC with reported works.

No.	Catalyst	Electrolyte	Potential (V vs. RHE)	TOF (s^{-1})	Ref.
1	Pt-Hg	0.1 M $HClO_4$	0.7	0.0839	18
			0.65	0.19	
			0.6	0.53	
2	Pd-Hg	0.1 M $HClO_4$	0.65	0.22561	19
			0.6	0.56098	
3	Ni-N_2O_2/C	0.1 M KOH	0.65	0.02	14
4	Mo_1/OSG-H	0.1 M KOH	0.65	0.076	20
5	O-CNT	0.1 M KOH	0.6	0.0178	21
6	F-mrGO	0.1 M KOH	0.65	0.088	22
7	COF-366-Ni	0.1 M KOH	0.65	0.33	23
			0.6	0.67	
8	ZnO_3C	0.1 M KOH	0.65	0.197	24
9	O-C(Al)	0.1 M NaOH	0.65	0.196	25
10	200-Pt-N-CNT	0.1 M $HClO_4$	0.6	0.5	26
11	Co-SCD-2	0.1 M KOH	0.6	0.044	27
12	BUCT-COF-7/CNT	0.1 M KOH	0.7	0.053	28
**13**	**Ni-SAC**	**0.1 M KOH**	**0.7**	**0.65**	**This work**
			**0.65**	**0.72**	
			**0.6**	**0.78**	

Supplementary Table 7. Mass activity of Ni-SAC compared to reported works.

No.	Catalyst	Electrolyte	Mass Activity ($A\ g^{-1}$) at 0.65 V	Ref.
1	O-CNTs	0.1 M KOH	20.6	21
2	Co-N-C	0.1 M KOH	12.1	29
3	$Ni_2Mo_6S_8$	0.1 M KOH	~ 0.93	30
4	In SAs/NSBC	0.1 M KOH	2.5	31
5	OCNS	0.1 M KOH	14.5	32
6	Co-POC-O	0.1 M KOH	16.5	33
7	N-FLG-8	0.1 M KOH	14.65	34
8	$GNP_{C=O,1}$	0.1 M KOH	8.87	35
9	O-C(Al)	0.1 M NaOH	28.5	25
10	Mo_1/-OSGH	0.1 M KOH	20.86	20
11	Ni-N_2O_2/C	0.1 M KOH	1.10	14
12	BUCT-COF-7/ CNT	0.1 M KOH	~11	28
13	Co-SCD-2	0.1 M KOH	~26.85	27
14	NBO-G/CNTs	0.1 M KOH	19.3	36
**15**	**Ni-SAC**	**0.1 M KOH**	**49.06**	**This work**

Supplementary Table 8. Comparison of $2e^-$ ORR performance in flow cell under alkaline conditions with currently reported work.

No.	Catalyst	Yield rate (mmol cm^{-2} h^{-1}) /(mol g_{cat}^{-1} h^{-1})	J_{max} (mA cm^{-2})	FE_{max}	Stability	Ref.
1	CNB-ZIL	0.447<br>1.787	133.3	~80%	9 h	37
2	NBO-G/CNTs	0.709<br>0.709	110	~81%	12 h	36
3	$OCNS_{900}$	0.770<br>0.770	100	~89.6%	11 h	32
4	Co-N-C	0.443<br>4.33	65	~68%	6 h	29
5	PANI/CDs-Co-2	0.875<br>3.5	~85	~86.5%	10 h	38
6	NiB_2	1.901<br>4.753	~250	~93%	12 h	39
7	Bi/PNC-4	0.276<br>2.76	~35	~60%	/	40
**8**	**Ni-SAC**	**2.19±0.02**<br>**5.48**	**~262**	**~91.4%**	**35 h**	**This work**

Supplementary Table 9. Average price references and corresponding sources for raw materials and products.

Chemicals	Price ($/kg)			Average	Source
O_2	0.035	0.14	0.1	0.092	https://app.indexbox.io/table/280440/0/, *Nat Commun.*, **2023**, 14, 6263
H_2O_2	1.2	1.3	0.76	1.087	https://www.chemanalyst.com/Pricing-data/hydrogen-peroxide-1169
EG	0.56	0.73	0.48	0.59	http://www.100ppi.com
GA	2.2	2.4	3.9	2.83	https://www.pharmacompass.com/price/glycolic-acid

Supplementary References

1. Tian Q, et al. Mesoporous carbon spheres with programmable interiors as efficient nanoreactors for H_2O_2 electrosynthesis. *Nat. Commun.* **15**, 983 (2024).
2. Jung E, *et al*. Atomic-level tuning of Co–N–C catalyst for high-performance electrochemical H_2O_2 production. *Nat. Mater.* **19**, 436-442 (2020).
3. Lee B-H, *et al*. Supramolecular tuning of supported metal phthalocyanine catalysts for hydrogen peroxide electrosynthesis. *Nat. Catal.* **6**, 234-243 (2023).
4. Buettner GR. Spin trapping: ESR parameters of spin adducts 1474 1528V. *Free Radical Bio. Med*. **3**, 259-303 (1987).
5. Fan K, *et al*. Active hydrogen boosts electrochemical nitrate reduction to ammonia. *Nat. Commun*. **13**, 7958 (2022).
6. Ji K, *et al*. Electrocatalytic hydrogenation of 5-Hydroxymethylfurfural promoted by a Ru_1Cu single-atom alloy catalyst. *Angew. Chem. Int. Ed.* **61**, e202209849 (2022).
7. Xu H, Zhang S, Geng J, Wang G, Zhang H. Cobalt single atom catalysts for the efficient electrosynthesis of hydrogen peroxide. *Inorg. Chem. Front*. **8**, 2829-2834 (2021).
8. Deng Z, *et al*. Electrocatalytic H_2O_2 production via two-electron O_2 reduction by Mo-doped TiO_2 nanocrystallines. *Catal. Sci. Technol.* **11**, 6970-6974 (2021).
9. Han L, *et al*. In-plane carbon lattice-defect regulating electrochemical oxygen reduction to hydrogen peroxide production over nitrogen-doped graphene. *ACS Catal.* **9**, 1283-1288 (2019).
10. Fu H, *et al*. Lattice strained B-doped Ni nanoparticles for efficient electrochemical H_2O_2 synthesis. *Small* **18**, 2203510 (2022).

11. Sun Y, *et al*. Structure, activity, and faradaic efficiency of nitrogen-doped porous carbon catalysts for direct electrochemical hydrogen peroxide production. *ChemSusChem* **11**, 3388-3395 (2018).

12. Wang M, *et al*. An Efficient interfacial synthesis of two-dimensional metal–organic framework nanosheets for electrochemical hydrogen peroxide production. *Angew. Chem. Int. Ed.* **60**, 11190-11195 (2021).

13. Chang Y, *et al*. Oxygenated boron-doped carbon via polymer dehalogenation as an electrocatalyst for high-efficiency O_2 reduction to H_2O_2. *Sci. China Mater*. **65**, 1276-1284 (2022).

14. Wang Y, *et al*. High-efficiency oxygen reduction to hydrogen peroxide catalyzed by nickel single-atom catalysts with tetradentate N_2O_2 coordination in a three-phase flow cell. *Angew. Chem. Int. Ed.* **59**, 13057-13062 (2020).

15. Li R, *et al*. Short-range order in amorphous nickel oxide nanosheets enables selective and efficient electrochemical hydrogen peroxide production. *Cell Rep. Phys. Sci.* **3**, 100788 (2022).

16. Shen J, Qiu X, Zhu Y. Nitrogen-doped sp^3 carbon dot catalysed two-electron electrochemical oxygen reduction for efficient production of hydrogen peroxide. *J. Mater. Chem. A* **11**, 11704-11711 (2023).

17. Guo Y, Xu Q, Yang S, Jiang Z, Yu C, Zeng G. Precise design of covalent organic frameworks for electrocatalytic hydrogen peroxide production. *Chem. Asian J.* **16**, 498-502 (2021).

18. Siahrostami S, *et al*. Enabling direct H_2O_2 production through rational electrocatalyst

design. *Nat. Mater.* **12**, 1137-1143 (2013).

19. Verdaguer-Casadevall A, *et al*. Trends in the electrochemical synthesis of H_2O_2: enhancing activity and selectivity by electrocatalytic site engineering. *Nano Lett.* **14**, 1603-1608 (2014).

20. Tang C, *et al*. Coordination tunes selectivity: two-electron oxygen reduction on high-loading molybdenum single-atom catalysts. *Angew. Chem. Int. Ed.* **59**, 9171-9176 (2020).

21. Lu Z, *et al*. High-efficiency oxygen reduction to hydrogen peroxide catalysed by oxidized carbon materials. *Nat. Catal.* **1**, 156-162 (2018).

22. Kim HW, *et al*. Efficient hydrogen peroxide generation using reduced graphene oxide-based oxygen reduction electrocatalysts. *Nat. Catal.* **1**, 282-290 (2018).

23. Liu C, *et al*. Intrinsic activity of metal centers in metal–nitrogen–carbon single-atom catalysts for hydrogen peroxide synthesis. *J. Am. Chem. Soc.* **142**, 21861-21871 (2020).

24. Jia Y, *et al*. Tailoring the electronic structure of an atomically dispersed zinc electrocatalyst: coordination environment regulation for high selectivity oxygen reduction. *Angew. Chem. Int. Ed.* **61**, e202110838 (2022).

25. Yang Q, *et al*. Atomically dispersed Lewis acid sites boost 2-electron oxygen reduction activity of carbon-based catalysts. *Nat. Commun.* **11**, 5478 (2020).

26. Zhao J, *et al*. Manipulating the oxygen reduction reaction pathway on Pt-coordinated motifs. *Nat. Commun.* **13**, 685 (2022).

27. Qi D, *et al*. Cyclodextrin-supported $Co(OH)_2$ clusters as electrocatalysts for efficient and selective H_2O_2 synthesis. *Angew. Chem. Int. Ed.* **62**, e202307355 (2023).

28. Zhang Y, *et al*. Multicomponent synthesis of imidazole-linked fully conjugated 3D covalent

organic framework for efficient electrochemical hydrogen peroxide production. *Angew. Chem. Int. Ed.* **62**, e202314539 (2023).

29. Sun Y, *et al*. Activity–selectivity trends in the electrochemical production of hydrogen peroxide over single-site metal–nitrogen–carbon catalysts. *J. Am. Chem. Soc.* **141**, 12372-12381 (2019).

30. Xia F, *et al*. Carbon free and noble metal free $Ni_2Mo_6S_8$ electrocatalyst for selective electrosynthesis of H_2O_2. *Adv. Funct. Mater.* **31**, 2104716 (2021).

31. Zhang E, *et al*. Engineering the local atomic environments of indium single-atom catalysts for efficient electrochemical production of hydrogen peroxide. *Angew. Chem. Int. Ed.* **61**, e202117347 (2022).

32. Chen S, *et al*. Chemical identification of catalytically active sites on oxygen-doped carbon nanosheet to decipher the high activity for electro-synthesis hydrogen peroxide. *Angew. Chem. Int. Ed.* **60**, 16607-16614 (2021).

33. Li B-Q, Zhao C-X, Liu J-N, Zhang Q. Electrosynthesis of hydrogen peroxide synergistically catalyzed by atomic Co–N_x–C sites and oxygen functional groups in noble-metal-free electrocatalysts. *Adv. Mater.* **31**, 1808173 (2019).

34. Li L, *et al*. Tailoring selectivity of electrochemical hydrogen peroxide generation by tunable pyrrolic-nitrogen-carbon. *Adv. Energy Mater.* **10**, 2000789 (2020).

35. Han G-F, *et al*. Building and identifying highly active oxygenated groups in carbon materials for oxygen reduction to H_2O_2. *Nat. Commun.* **11**, 2209 (2020).

36. Fan M, *et al*. N–B–OH site-activated graphene quantum dots for boosting electrochemical hydrogen peroxide production. *Adv. Mater.* **35**, 2209086 (2023).

37. Tian Z, *et al*. Constructing interfacial boron-nitrogen moieties in turbostratic carbon for electrochemical hydrogen peroxide production. *Angew. Chem. Int. Ed.* **61**, e202206915 (2022).

38. Zhou Y, *et al*. Efficient synthesis of H_2O_2 via oxygen reduction over PANI driven by kinetics regulation of carbon dots. *Appl. Catal. B: Environ.* **322**, 122105 (2023).

39. Wu J, *et al*. Composition engineering of amorphous nickel boride nanoarchitectures enabling highly efficient electrosynthesis of hydrogen peroxide. *Adv. Mater.* **34**, 2202995 (2022).

40. Bao Z, *et al*. Tuning the ratio of Bi/Bi_2O_3 in Bi/PNC nanosheet for high-efficiency electrosynthesis hydrogen peroxide. *Nano Res.* **16**, 9050-9058 (2023).
